# Deflective and intimidating eyespots: a comparative study of eyespot size and position in *Junonia* butterflies

**DOI:** 10.1002/ece3.831

**Published:** 2013-10-16

**Authors:** Ullasa Kodandaramaiah, Patrik Lindenfors, Birgitta S Tullberg

**Affiliations:** 1 School of Biology, Indian Institute of Science Education and Research ThiruvananthapuramThiruvananthapuram, 695 016, India; 2 Department of Zoology, University of StockholmSE-10691, Stockholm, Sweden

**Keywords:** Butterflies, deflection, eyespots, intimidation, *Junonia*, *Junonia almana*

## Abstract

Eyespots are conspicuous circular features found on the wings of several lepidopteran insects. Two prominent hypotheses have been put forth explaining their function in an antipredatory role. The deflection hypothesis posits that eyespots enhance survival in direct physical encounters with predators by deflecting attacks away from vital parts of the body, whereas the intimidation hypothesis posits that eyespots are advantageous by scaring away a potential predator before an attack. In the light of these two hypotheses, we investigated the evolution of eyespot size and its interaction with position and number within a phylogenetic context in a group of butterflies belonging to the genus *Junonia*. We found that larger eyespots tend to be found individually, rather than in serial dispositions. Larger size and conspicuousness make intimidating eyespots more effective, and thus, we suggest that our results support an intimidation function in some species of *Junonia* with solitary eyespots. Our results also show that smaller eyespots in *Junonia* are located closer to the wing margin, thus supporting predictions of the deflection hypothesis. The interplay between size, position, and arrangement of eyespots in relation to antipredation and possibly sexual selection, promises to be an interesting field of research in the future. Similarly, further comparative work on the evolution of absolute eyespot size in natural populations of other butterfly groups is needed.

## Introduction

Wings of butterflies in the genus *Junonia* (Nymphalidae: Nymphalinae) exhibit a stunning array of conspicuous circular patterns called eyespots (Kodandaramaiah 2009). The evolutionary significance of eyespots is a highly debated topic, with hypotheses ranging from contexts of sexual selection (Oliver et al. 2009; Prudic et al. 2011) and species recognition to that of adaptation against predation (Stevens 2005; Kodandaramaiah 2011 and references therein). Within *Junonia*, features of eyespots such as their number, arrangement, size, and coloration vary markedly across species, at the same time being conserved within species. Kodandaramaiah (2009) studied the evolution of eyespot patterning across the phylogeny of the group and concluded that diverse selective forces across the phylogeny have shaped eyespot morphology in this group. We here test predictions related to the role of eyespots against predation in *Junonia*.

Two major hypotheses have been postulated with regard to eyespots in a defense context – the “intimidation” and “deflection” hypotheses (reviewed in Stevens 2005 and Kodandaramaiah 2011). The intimidation hypothesis posits that large, conspicuous eyespots scare predators either by (1) mimicking eyes of the predators’ own potential predators (the eye-mimicry hypothesis; Blest 1957); or by (2) being highly conspicuous features *per se* (Blest 1957; Stevens 2005). Although there is an ongoing debate about the relative importance of the two mechanisms of intimidation (Stevens et al. 2008a; Merilaita et al. 2011; Blut et al. 2012), for the purpose of this study, it suffices to mention that accumulating experimental evidence strongly indicates that eyespots can thwart potential predatory attacks (Vallin et al. 2005, 2007; Stevens et al. 2007, 2008a,b; Kodandaramaiah et al. 2009; Merilaita et al. 2011; Blut et al. 2012; Olofsson et al. 2013a). Of particular relevance to this article is the study by Kodandaramaiah et al. (2009), which showed that the large and conspicuous eyespots of *Junonia almana* inhibit attacks by birds.

Predators often strike prey at the most vulnerable parts, such as the head and thorax, in order to immobilize it (cf. Olofsson et al. 2010). The deflection hypothesis predicts that marginal eyespots increase survival because a predatory attack is redirected toward the eyespots, and therefore, the butterfly has time to escape, albeit with a slightly damaged wing (Poulton 1890). The ventral surface of many butterflies is seasonally polymorphic, where the relatively inactive dry season form lacks eyespots and is cryptic, while the wet season form has putative marginal eyespots that are thought to be deflective (Brakefield and Larsen 1984). Evidence from several studies support predictions of the deflection hypothesis (Wourms and Wasserman 1985; Lyytinen et al. 2004, Olofsson et al. 2010, 2013b; Vallin et al. 2011; Sourakov 2013).

**Figure 1:**
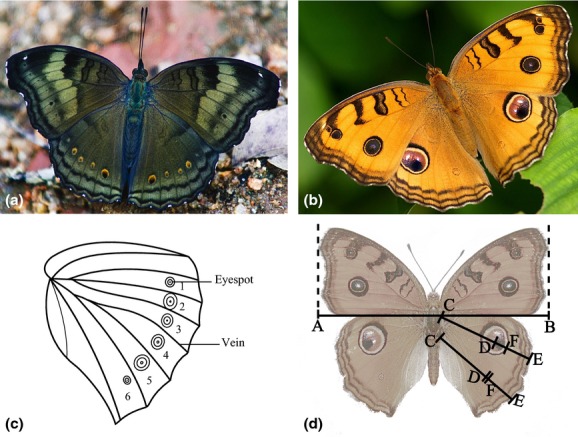
Illustrations depicting eyespot patterns and measurements used in the analyses. (a) Example of a “serial” eyespot arrangement – *Junonia iphita* (b) Example of an “individual” eyespot arrangement – *Junonia almana*. Note that eyespots 1 and 2 are fused to form a large, composite eyespot in this species. (c) Illustration of wing venation with the eyespot numbering system used here. The area bounded by veins is referred to as the wing compartment. (d) Measurements taken: wingspan (A–B); distance from thorax to wing margin, that is, “hindwing length” (C–E); distance from thorax to eyespot center (C–D). The eyespot diameter was measured along CE. The distance from the eyespot to the wing margin was either DE or FE.

In *Junonia*, there are six wing compartments, that is, areas bounded by veins, which can contain an eyespot. Eyespots are found in two basic configurations (Fig. 1): (1) Serial, where eyespots in each compartment together form a row (Fig. 1a); and (2) Solitary or individual, where two to five compartments lack eyespots (Kodandaramaiah 2009; Fig. 1b). Kodandaramaiah (2009) showed that both solitary and serial configurations have evolved multiple times over the phylogeny of *Junonia* and related butterflies, with switches in both directions. In the light of the two aforementioned hypotheses, we here formulate and test predictions about the evolution of serial and solitary eyespots in this genus.

The effectiveness of an intimidating eyespot is expected to increase with size and enhanced conspicuousness (Stevens et al. 2007, 2008a). Kodandaramaiah (2009) suggested that in *Junonia*, intimidating eyespots are more likely to be solitary. This is because a solitary configuration allows an eyespot to (1) be larger by extending into adjacent compartments not adorned with eyespots; and (2) “stand out” better on the wing surface as compared to being a serial configuration, and thus appear more conspicuous. We hence predict that eyespots in solitary configurations are larger compared with those in serial configurations.

Although there is no clear prediction from the deflection hypothesis with respect to whether deflective eyespots should be solitary or serial, such eyespots are thought to be smaller than intimidating eyespots (Stevens 2005). Moreover, selection for a deflective effect should favor placement of the eyespot closer to the wing margin such that a deflected attack (1) is farther away from vital body parts; and (2) results in lesser damage to wing tissue. We therefore predict a negative relationship between eyespot size and proximity to wing margin.

## Material and Methods

### Phylogeny and data collection

In this article, we have defined an eyespot as a round formation that consists of a disc, that is, encircled by at least one complete ring.

We used a phylogeny of *Junonia* from a reconstruction of the tribe Junoniini based on DNA sequence data (Kodandaramaiah 2009). Maintaining consistency with Kodandaramaiah et al. (2009), we restrict our analysis to the dorsal hindwing. Eyespots on the ventral surface in many *Junonia* species are seasonally plastic or highly reduced (Blest 1957; Kodandaramaiah 2009), and the dorsal hindwing displays the best developed eyespots in almost all species. However, we stress that eyespot configurations with respect to number and position in *Junonia* tend to be conserved both dorsoventrally and across the fore- and hindwings, unlike in other groups such as the mycalesines, which includes *Bicyclus anynana*, the model species in which eyespots have been extensively studied (Brakefield 2010). In mycalesines, each wing surface can have an unique eyespot configuration.

*Junonia* species have one to six eyespots (Fig. 1a, b and c). We measured representatives of each species from butterfly handbooks (D'Abrera 1982, 1990, 1997; Scott 1986; Pringle et al. 1994) using a ruler and a calliper. In cases of sexual dimorphism (known in *Junonia orithya* and *Junonia hierta*) and/or where both sexes were depicted, we used the female. This is because we test predictions related to natural selection and the chance of the wing pattern being a product of sexual selection, for example mate choice, is higher for males (Wiernasz 1989; Robertson and Monteiro 2005; Kemp 2007).

The measurements were used directly when photographs represented life size. When photographs were smaller, measurements were scaled to correspond to life size. We measured the diameter of the eyespot in compartments 2 and 5 (Fig. 1c and d). These eyespots were chosen because they are present in almost all *Junonia* species, whereas other eyespots are more sporadic in occurrence (see Kodandaramaiah 2009). For an estimate of the wingspan of the butterfly, we measured the total span between the forewings as depicted in Figure 1D. In order to get a measurement of the position of an eyespot, we measured (1) the distance from the thorax to the center of the eyespot, that is, “CD” in Figure 1D; and (2) the distance between the thorax and the wing margin measured through the eyespot, that is, “CE” in Figure 1D. Both these measurements were taken parallel to the veins surrounding the compartment with the eyespot (in the cases where eyespots spanned two compartments, for instance in *Junonia almana,* measurements were taken along the vein bisecting the eyespot). We divided the first value with the second and multiplied the quotient by 100, which provides an index of the position of the eyespot as a percentage of the distance from thorax to wing margin. We also calculated the index of position using the distance between the distal edge of the eyespot and the wing margin, that is, “CF” in Figure 1D.

### Data analysis

Phylogenetic independent contrast and matched pairs analyses were carried out using Mesquite 3.0 (Maddison and Maddison 2011). The relationship between size and position of eyespots in compartments 2 and 5 was investigated with phylogenetic paired *t*-tests (Lindenfors et al. 2010) using the Phytools package (Revell 2011) and phylogenetic means calculated using the APE package (Paradis et al. 2004), both in R. Branch length transformations did not yield any improvements according to diagnostics as described by Garland et al. (1992). Therefore, all branch lengths were set to equal length. Analyses that include eyespot distance from wing margin were performed on measurements based on both eyespot center and eyespot edge.

## Results

Of the 22 *Junonia* species investigated, 18 have eyespots in both compartments, one species has an eyespot only in compartment 2, and three species have an eyespot only in compartment 5. Eyespot size ranges from 1 to over 8 mm for compartment 2, and the phylogenetic average size is larger, but not significantly so, for compartment 2 (phylogenetic mean = 5.37, 95% confidence interval = 4.13–6.80) than for compartment 5 (phylogenetic mean = 3.48, 95% confidence interval = 2.74–4.22; *t* = 1.65, df = 17, *P* = 0.119, phylogenetic paired *t*-test).

All investigated eyespots are positioned closer to the wing margin than to the thorax, but there was no significant difference in the phylogenetic average position for compartments 2 (71.49%) and 5 (74.46%; *t* = 1.598, df = 17, *P* = 0.131, phylogenetic paired *t*-test). Results were similar when positions were based on eyespot edges (*x* = 76.13% for compartment 2 and *x* = 77.44% for compartment 5; *t* = 0.761, df = 17, *P* = 0.458).

In analyses based on phylogenetic contrasts, we found no significant correlation between eyespot size and butterfly wingspan (compartment 2: *P* = 0.213, df = 17; compartment 5: *P* = 0.119, df = 19). We also found no significant correlation between eyespot size and hindwing size (compartment 2: *P* = 0.608, df = 17; compartment 5: *P* = 0.780, df = 19). Thus, there is no indication that eyespot size variation in either compartment under study is dependent on body size, and therefore, we see no reason to adjust eyespot size to body size. Neither did we find a significant correlation between the size of eyespots in the two compartments (*P* = 0.203, df = 16), which indicates independent size variation for the two eyespots.

**Figure 2:**
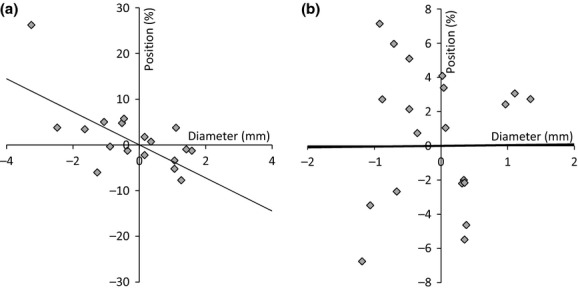
The correlation between phylogenetic contrasts of eyespot size and position for (a) compartment 2 (regression through the origin b = −3.164, *t* = 3.769, df = 17, *P* = 0.002) and (b) compartment 5 (regression through the origin b = 0.042, *t* = 0.034, df = 19, *P* = 0.973).

We found a significant negative correlation between size and position of the eyespot in compartment 2 (regression through the origin b = −3.164, *t* = 3.769, df = 17, *P* = 0.002; Fig. 2a) but no significant relationship between size and position for compartment 5 (regression through the origin b = 0.042, *t* = 0.034, df = 19, *P* = 0.973; Fig. 2b). Results were similar when analyses were based on the position of eyespot edges (compartment 2: regression through the origin b = −2.285, *t* = −2.415, df = 17, *P* = 0.027; compartment 5: regression through the origin b = 0.245, *t* = 1.165, df = 19, *P* = 0.870). Thus, for compartment 2, we found that smaller eyespots are positioned closer to the wing margin.

**Figure 3:**
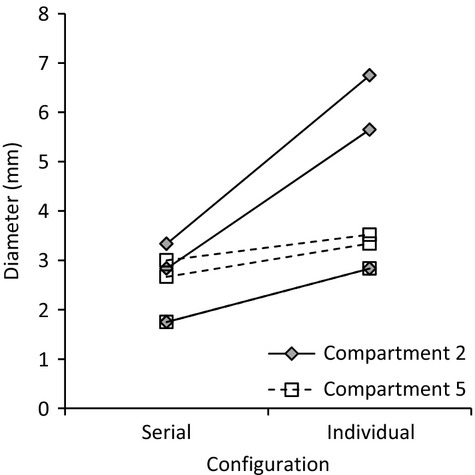
Matched pairs comparison of eyespot size between individual and serial configurations for compartment 2 (individual *x* = 5.08, SD = 2.02; serial *x* = 2.64, SD = 0.81; *t* = −3.49, df = 2, *P* = 0.073, paired *t*-test) and compartment 5 (individual *x* = 3.23, SD = 0.36, serial *x* = 2.47, SD = 0.65; *t* = −4.51, df = 2, *P* = 0.046, paired *t*-test; *N* = 3 for each compartment).

We found three phylogenetic matched pairs between the serial and individual configurations in *Junonia*. Individual eyespots were larger than serial eyespots in each matched pair (Fig. 3), the analyses showing a statistical trend for compartment 2 (individual *x* = 5.08, SD = 2.02; serial *x* = 2.64, SD = 0.81; *t* = −3.49, df = 2, *P* = 0.073, paired *t*-test), and a significant difference for compartment 5 (individual *x* = 3.23, SD = 0.36, serial *x* = 2.47, SD = 0.65; *t* = −4.51, df = 2, *P* = 0.046, paired *t*-test; Fig. 3).

**Figure 4:**
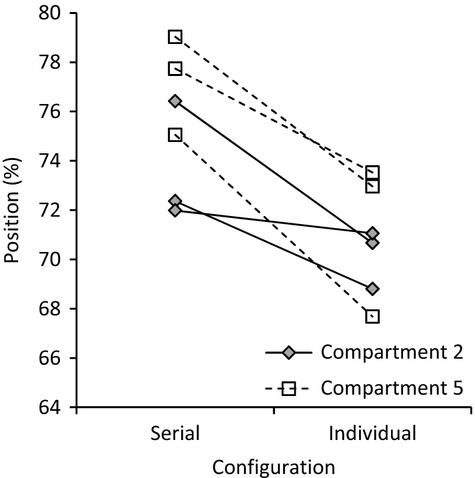
Matched pairs comparison of eyespot position between individual and serial configurations for compartment 2 (individual *x* = 70.18, SD = 1.21; serial *x* = 73.59, SD = 2.46; *t* = 2.44, df = 2, *P* = 0.135, paired *t*-test) and compartment 5 (individual *x* = 71.39, SD = 3.23, serial *x* = 77.27, SD = 2.03; *t* = 6.41, df = 2, *P* = 0.024, paired *t*-test). (*N* = 3 for each compartment).

In each matched pair, serial eyespots were positioned closer to the margin than individual eyespots (Fig. 4), but the relationship was statistically significant only for compartment 5 (compartment 2: individual *x* = 70.18, SD = 1.21; serial *x* = 73.59, SD = 2.46; *t* = 2.44, df = 2, *P* = 0.135, paired *t*-test; compartment 5: individual *x* = 71.39, SD = 3.23, serial *x* = 77.27, SD = 2.03; *t* = 6.41, df = 2, *P* = 0.024, paired *t*-test; Fig. 4). Results were similar when analyses were based on the position of eyespot edges (compartment 2: individual *x* = 75.03, SD = 0.78; serial *x* = 76.24, SD = 3.17; *t* = 0.84, df = 2, *P* = 0.491, paired *t*-test; compartment 5: individual *x* = 74.59, SD = 2.78, serial *x* = 79.76, SD = 2.01; *t* = 11.31, df = 2, *P* = 0.008, paired *t*-test).

## Discussion

*Junonia* is an ideal group to study the relationships between eyespot size and position. There is knowledge of sister-group relationships among species and the evolution of serial and solitary eyespots over the phylogeny. Furthermore, given the multiple switches between serial and solitary eyespots, the group presents an unique opportunity to test the effects of eyespot disposition (in terms of being serial or solitary) on size and position.

### Prediction 1: Solitary eyespots are larger than serial eyespots

As predicted, solitary eyespots are larger than corresponding serial eyespots on the same compartment. This effect was significant for compartment 5, whereas for compartment 2 there is a trend in the same direction. Thus, the results suggest selection for larger eyespot size when eyespots are solitary, but not when they are arranged serially. In some species such as *J. almana* and *Junonia coenia*, eyespots of two compartments have even suffused to one larger twin eyespot. Given the multiple lines of evidence for an intimidating effect of eyespots in butterflies, we opine that selection for an intimidation function in *Junonia* is the most likely explanation for why solitary eyespots tend to be larger.

However, we do not discount the possibility of sexual selection for larger eyespot size. There have been no studies testing the function of these eyespots in sexual selection. In *Bicyclus anynana*, the central UV-reflective pupils of solitary eyespots on the dorsal surface are thought to be under sexual selection (Robertson and Monteiro 2005; Oliver et al. 2009; Prudic et al. 2011). Females of this species have been shown to select males based on the size of the pupils, which appears to have resulted in stabilizing selection for the size of the pupils. In the light of this, studies are needed to ascertain whether eyespot size in *Junonia* is selected during courtship.

### Prediction 2: There is a negative relationship between eyespot size and proximity to wing margin

For eyespots on compartment 2, we found a direct negative correlation between size and proximity to wing margin. Furthermore, there was indirect support for this prediction from compartment 5; on this compartment, lineages with a serial configuration had eyespots that were both significantly smaller and were placed significantly closer to the margin than compared with lineages with solitary eyespots.

These results are consistent with the idea that smaller eyespots are selected for a deflective effect. It is important to note that the results do not change even when we calculated the position based on the distance of the eyespot margin from the wing margin. As in the case of solitary eyespots, we cannot rule out sexual selection on serial eyespots. However, sexual selection *per se* does not satisfactorily explain why smaller eyespots are found closer to the wing margin compared with larger ones.

We recognize that the use of limited samples based on books, as in the current study and in Kodandaramaiah (2009), is not optimal. However, we argue that in this particular study, the results will not change with a higher number of measurements per species, unless there is a systematic bias in the books favoring our predictions. For instance, if books have selectively illustrated photographs of specimens with eyespots closer to the wing margin in the case of species with smaller eyespots but not for species with larger eyespots. Such a scenario is highly unlikely. We also acknowledge that we have not been able to account for intraspecific variation among populations, within populations or between sexes. Such variation can be quite significant in some species of butterflies. Profound intraspecific variation is possibly due to the interplay between a deflective and intimidating function in some species. However, our inferences about the position of eyespots in relation to size in *Junonia* is unlikely to change with improved sampling. Our results form the basis for a more comprehensive study across butterflies utilizing museum or natural samples to take into account intraspecific variation.

**Figure 5:**
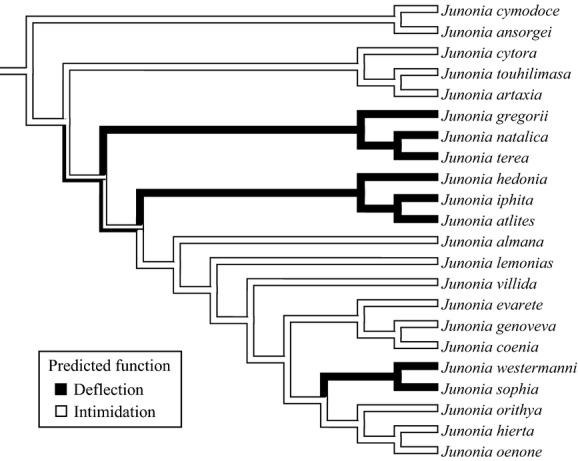
Predicted functions of dorsal hindwing eyespots in *Junonia* species mapped on to their phylogeny. Serial and solitary eyespots are assumed to be deflective and intimidating, respectively. The phylogeny and reconstruction of the evolution of serial versus solitary are redrawn from Figure 4 of Kodandaramaiah (2009).

Under the assumption that serial eyespots are used in deflection, and solitary eyespots in intimidation, it appears that both deflective and intimidation functions of eyespots have evolved more than once in *Junonia* (Fig. 5). Closely related sympatric species pairs with deflective and intimidating eyespots, respectively, will be especially interesting as study organisms to understand the evolution of these two kinds of eyespots. For instance, *Junonia almana*, *Junonia lemonias*, *Junonia iphita,* and *Junonia atlites* occur sympatrically in many parts of Asia. *J. almana* and *J. lemonias* have solitary eyespots, whereas the other two possess serial eyespots.

Wing compartment size presumably varies in direct relation to body size, and because eyespots are generally present within a compartment, we expected eyespot size to be correlated with wing size. Surprisingly, there was no significant correlation between eyespot size and wingspan. This suggests that eyespot size evolution in *Junonia* is relatively free from constraints of general body size. Furthermore, we found no correlation between sizes of eyespots in the two compartments, supporting the idea that selection on eyespot size in individual compartments is not strongly constrained by developmental underpinnings (Beldade et al. 2002).

## Summary and Conclusion

This is the first study investigating the evolution of eyespot size and its interaction with position and number within a phylogenetic context. We found that larger eyespots tend to be found individually, rather than in serial dispositions. Larger size and conspicuousness make intimidating eyespots more effective, and we suggest that our results support an intimidation function in some species of *Junonia* with solitary eyespots. Our results also show that smaller eyespots in *Junonia* are located closer to the wing margin, thus supporting predictions of the deflection hypothesis. The interplay between size, position, and arrangement of eyespots in relation to defense from predators, and possibly sexual selection, promises to be an interesting field of research in the future. Similarly, further comparative work on the evolution of absolute eyespot size in natural populations of other butterfly groups is needed.
